# Discrimination of different cancer types clustering Raman spectra by a super paramagnetic stochastic network approach

**DOI:** 10.1371/journal.pone.0213621

**Published:** 2019-03-12

**Authors:** JL González-Solís

**Affiliations:** Biophysics and Biomedical Sciences Laboratory, Centro Universitarios de los Lagos, Universidad de Guadalajara, Lagos de Moreno, Jalisco, Mexico; University of Missouri Columbia, UNITED STATES

## Abstract

Based in high sensitivity and specificity reported recently in detection of the cancer, the technique of Raman spectroscopy is proposed to discriminate between breast cancer, leukemia and cervical cancer using blood serum samples from patients officially diagnosed. In order to classify Raman spectra, clustering method known as Super Paramagnetic Clustering based on statistical physics concepts with a stochastic approach was implemented. Comparing firstly average Raman spectra of the three cancers, some peaks that allowed differentiating one cancer from other were identified, however, other peaks allowed concluding that there are biochemical similarities among them. According to these spectra, the band associated with amide I (1654 *cm*^−1^) and one of two shoulders assigned to amide III (1230-1282 *cm*^−1^) allowed discriminating leukemia from breast and cervical cancer, whereas band 714 *cm*^−1^ (polysaccharides) achieves to differentiate cervical cancer from leukemia and breast cancer, and bulged region, 1040 − 1100 *cm*^−1^ (phenylalanine, phospholipid) discriminated breast cancer from leukemia and cervical cancer. Subsequently, Super Paramagnetic Clustering method was applied to Raman spectra to study similarity relationships between cancers based on the biochemical composition of serum samples. Finally, as a cross check method, the standard method to classify Raman spectra of breast cancer, leukemia and cervical cancer, known as principal components analysis, was used showing excellent agreement with results of Super Paramagnetic Clustering method. Preliminary results demonstrated that Raman spectroscopy and Super Paramagnetic Clustering method can be used to discriminate between breast cancer, leukemia and cervical cancer samples using blood serum samples.

## Introduction

Although some of the most deadly cancers affect different parts of the body, among them there are similarities of different types according to several reported researches, ie, these studies had found relationships between certain forms of breast, lung, colon, cervical cancers and leukemia. The best knowledge of these similarities based on the same molecular origin could facilitate the comparison of therapeutic data bank between the cancers difficult to treat, suggesting that the treatments could be performed with the same chemotherapy drugs.

In this sense, studies have showed that the human cervical cancer oncogene (HCCR) is not only over-expressed in human cervical cancer tissues but also found to have high-level expression in various human malignancies including breast, kidney, stomach, colon, liver and ovarian cancer [1–6]. In addition, although several published data have shown that HCCR expression in some solid tumors is correlated to clinical outcome and confirmed it as a good biomarker of monitoring disease progression, its specific role in leukemia remained elusive [4–6]. Nevertheless, later studies were able to show that expression levels of the HCCR mRNA are associated with clinical prognosis in patients with acute leukemia (AL) and they have explored the potential use as a biomarker for monitoring minimal residual disease (MRD) in AL [7].

On the other hand, human papilloma viruses (HPVs) are accepted as being carcinogenic in human cervical and anogenital cancers. The suspicion that HPVs may also have a role in human breast cancer is based on the identification of HPVs in human breast tumours and the immortalisation of normal human breast cells by HPV types 16 and 18 [8, 9]. In addition, Kan *et al* extracted DNA from 50 unselected invasive ductal breast cancer samples by polymerase chain reaction (PCR) for HPV type 16, 18 and 33 gene sequences and showed that HPV 18 gene sequences are present in DNA extracted from breast tumours in Australian women [10]. Therefore, human papilloma viruses could also play a important role in human breast cancer.

In addition, several studies have reported that patients treated with adjuvant chemotherapy regimens, commonly including alkylating agents and anthracyclines, are at increased risk of developing leukemia, further enhanced by the use of radiotherapy [11].

In this work, the similarities between cancers based on the biochemical composition of blood serum samples from breast cancer, leukemia and cervical cancer patients were studied. The technique of Raman spectroscopy was used to know the biochemical composition of the samples and the Super Paramagnetic Clustering (SPC) method to discriminate between the samples from patients with the different cancer types that were studied. As a method of cross check tried to discriminate the Raman spectra using the standard method of classification of Raman spectra known as principal component analysis (PCA) observing the same main clusters obtained with SPC method.

Raman spectroscopy is based on the measurement of the vibrational energy levels of chemical bonds by measuring the inelastically scattered light following excitation. Therefore, Raman spectroscopy is a non-destructive analytical technique that provides fingerprint spectra with spatial resolution of an optical microscope with less or no sample preparation. It is fast emerging as a promising alternative technique in biology and medicine, including in the diagnosis of a variety of degenerative diseases such as cervical and breast cancer using serum samples with high sensitivity and specificity [12, 13]. Furthermore, monitoring of leukemia chemotherapy treatment using Raman spectroscopy was possible through the exclusive use of blood serum samples [14]. The results of cancer detection are markedly improved when the Raman technique is implemented with gold or silver nanoparticles, a technique known as Surface Enhanced Raman Spectroscopy (SERS) technique [15, 16]. In all these detection works, the Raman spectra are analyzed using PCA.

Extraction of meaningful information from Raman spectra data is a complex task due to the large volume of data and the expected complexity of its structure and organization. In order to address this problem several specialized methods, known as clustering techniques, have been developed during the past few years [17–19]. All these techniques, though differing in details, in essence try to identify patterns in data whose members have similar characteristics. Among the clustering techniques that employ concepts of statistical physics with a stochastic approach successfully tested on a variety of data sets [20] is the clustering method known as SPC [18]. This method exploits the properties of phase transitions in disordered Potts ferromagnets. The SPC method has already been used as an alternative method to PCA in cancer detection with excellent agreement between both results [21].

In the present article, firstly SPC method is briefly explained, followed by its application to classification of the Raman spectra of the blood serum samples from patients with the different cancer types studied. Finally, comparison with the results obtained using PCA is discussed.

## Materials and methods

### Serum samples from cancer patients

Serum samples were obtained from seven patients who were clinically diagnosed with leukemia, 3 acute lymphoblastic leukemia (ALL) patients, 2 acute myeloid leukemia (AML) patients and 2 chronic myeloid leukemia (CML) patients. No chronic lymphoblastic leukemia (CLL) case was registered. The age for the leukemia patients was between 8 and 50 years. Leukemia serum sample were provided by Hospital Regional de Alta Especialidad del Bajío from León city.

In addition to leukemia patients, serum samples from 3 and 19 patients, who were clinically diagnosed as cervical intraepithelial neoplasia I and squamous cervical carcinoma (SCC) respectively, were considered for this study. Results for colposcopy are reported by the pathologist as cervical intraepithelial neoplasia (CIN), carcinoma in situ (CIS), or squamous cervical carcinoma (SCC). CIN precancerous changes are categorized according to severity, CIN I, CIN II, and CIN III. CIN III is considered the same as carcinoma in situ (CIS) or stage 0 cervical cancer. At the time of diagnosis, age for the two cervical cancer patient groups was between 20 and 55 years.

Finally, serum samples from 16 patients who were clinically diagnosed with breast cancer were analyzed. Cervical and breast cancer serum samples were provided by Centro de Investigación Biomédica de Occidente from Guadalajara city.

None of the patients were under chemotherapy cancer treatment. All patients were from the central region of Mexico and had similar ethnic and socioeconomic backgrounds. The approval for the development of the protocols was authorized by the Ethical and National Health Research Committee of the Instituto Mexicano del Seguro Social (IMSS), with registration numbers R-2009-785-082 and R-2012-785-090. Written informed consent from healthy volunteers and patients with cancer (in compliance with the Helsinki Declaration) was also required and obtained prior to blood sample collection.

### Raman measurements

Blood samples were obtained between 7:00 and 9:00 a.m. and were centrifuged to get the serum. A drop of serum was placed onto an aluminum substrate, which was examined by an Olympus microscope integrated to the Raman system. All spectra were measured on the same day when the samples were obtained. In order to ensure statistically sound sampling, at least 5 spectra from different regions of each fresh serum sample were collected using a Horiba Jobin Yvon LabRAM HR800 confocal Raman microscope with a laser of 830 *nm* and 17 *mW* of power. The laser beam was focused on the surface of the sample with a 50× microscope objective and an exposure of 40 to 60 seconds. Analyzed region was from 400 to 1, 800 *cm*^−1^, with a spectral resolution of ∼ 0.6 *cm*^−1^. The Raman system was calibrated with a silicon semiconductor using the Raman peak at 520 *cm*^−1^.

Ten spectra per breast cancer patient were measured obtaining a total of 160 breast cancer spectra. In addition to breast cancer spectra, a total of 150 spectra were collected with 18 spectra from 3 *CIN* *I* patients and 132 spectra from 19 SCC patients. Finally, 42 spectra of leukemia patients were measured, 31 spectra of ALL, 6 of AML and 5 of CML. Details of the samples used in the study are shown in Table 1.

**Table 1:** Details of serum samples used in the study.

Spectrum Number	Nature	No. Patients
1-160	Breast cancer	16
161-310	Cervical cancer	21
311-352	Leukemia	7

### Super Paramagnetic Clustering method

We place a Potts spin variable *s*_*i*_ at each point *v*_*i*_. The spins at nearest neighboring points *v*_*i*_ and *v*_*j*_ couple via a short range ferromagnetic interaction *J*_*ij*_,

$$J_{ij}=J_{ji}=\frac{1}{\hat{K}}exp\{-\frac{1}{2}{\left(\frac{d_{ij}}{\bar{d}}\right)}^{2}\},$$ (1)

where *d*_*ij*_ is the Euclidean distance between points *v*_*i*_ and *v*_*j*_, $\bar{d}$ is the mean distance between interacting neighbors, and $\hat{K}$ is the average number of interacting neighbors of a point [22–24]. *J*_*ij*_ is some positive monotonically decreasing function of the distance, *d*_*ij*_, so that the closer two points are to each other, the more they like to belong to the same class. The interaction between spins that are not neighbors is set to zero.

The energy function of the system is governed by the Hamiltonian of an inhomogeneous ferromagnetic Potts model

$$H=\sum_{\left(i,j\right)}^{}J_{ij}\left(1-\delta_{s_{i},s_{j}}\right)\;s_{i}=1,2,...,q$$ (2)

where the summation is over interacting neighbors and delta function $\delta_{s_{i},s_{j}}=1$ if *s*_*i*_ = *s*_*j*_ and zero otherwise.

Disordered Potts systems may have the three different phases; ferromagnetic, paramagnetic, and superparamagnetic, depending on the temperature and interactions. The system is ferromagnetic at low temperatures *T* and paramagnetic at high. On increasing the temperature from zero, the system passes from a ferromagnetic to a paramagnetic state either directly in a single transition or via an intermediate superparamagnetic phase. This last via is of considerable interest in the study of disordered systems, especially in the analogy with data clustering as clusters of aligned spins automatically divide the data into its natural classes and a clear hierarchical structure among the classes emerges on varying the temperature.

The SPC method defines the clusters based on the collective behavior of the system instead of the simple distance between points through the average spin-spin correlation function, $g_{ij}=\langle \delta_{s_{i},s_{j}}\rangle$. This function is used to decide whether or not two spins belong to the same cluster.

**Fig 1:**
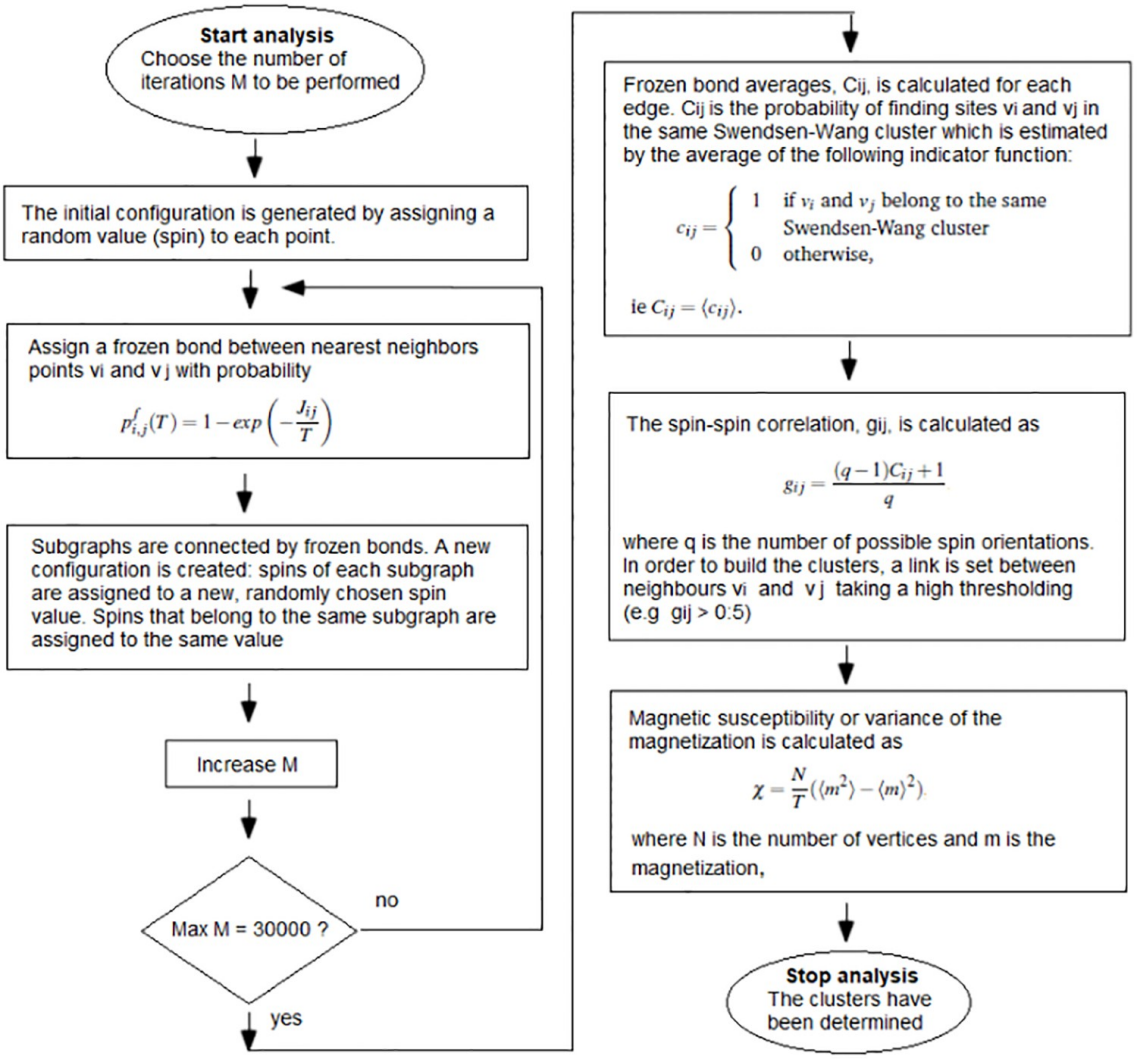
Schematic form of the implementation of the SPC algorithm.

The SPC technique as described by Blatt [23, 24] is apply. Blatt *et al* used the Swendsen-Wang method [17, 25] for the Markov chain Monte Carlo Simulation of Potts model with the procedure described by González *et al* [21]. This procedure allows determining the Swendsen-Wang clusters using the frozen bonds, calculate the spin-spin correlation [18] taking a high thresholding (e.g *g*_*ij*_ > 0.5) and finally, calculate the magnetic susceptibility, *χ*, [19]. When the temperature is low and it begins to increases quickly then clusters begin to split. As the temperature is raised, the system may break first into two clusters, each of which breaks into more subclusters and so on. Such a hierarchical structure of the magnetic clusters reflects a hierarchical organization of the data into categories and subcategories. A schematic form of the implementation of the SPC algorithm is shown in Fig 1.

**Fig 2:**
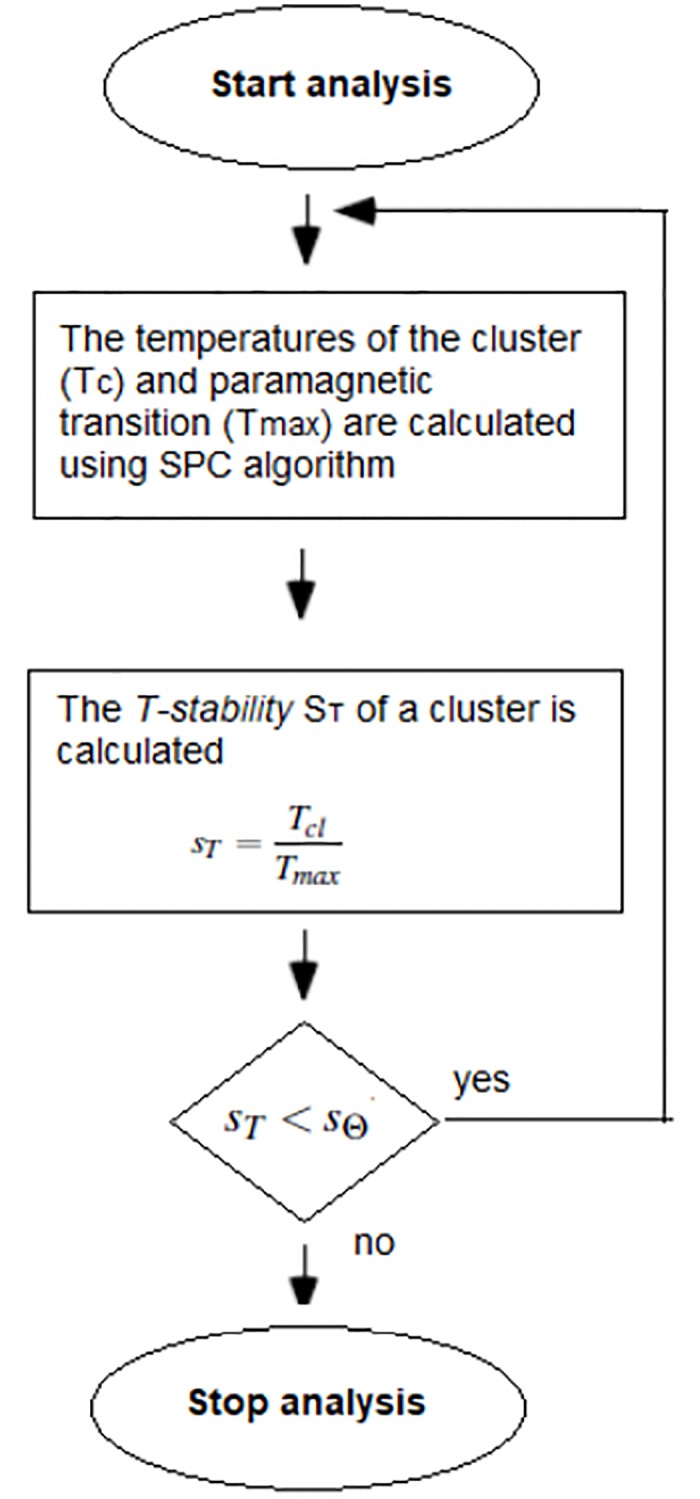
Schematic form of the sequential algorithm.

Once the clusters have been determined, the most natural (without substructures) and stable clusters are determined. Natural clusters express themselves as regions of order that are stable over an entire phase, ie, over a substantial range of *T*. For this reason, Ott *et al* proposed to choose as the most natural and stable clusters those that have the largest *T-range* extensions (denoted by *T*_*cl*_) [20] and are less stable than a threshold value *s*_Θ_ (*s*_*T*_ < *s*_Θ_), where *s*_*T*_ expresses the stability of the cluster in relation to the stability of the whole set. This threshold value, *s*_Θ_, is the main control parameter that is set from outside. In order to find the best clusters (the most natural and stable), they introduced a sequential procedure [26] (see Fig 2).

In order to extract the meaningful information from cancer Raman spectra, so as its structure and organization, this clustering technique, novel in the research areas of spectroscopy, will be used. The technique in essence attempts to identify patterns in the bank of spectra whose members have similar spectral characteristics.

For its application to the bank of spectra, first a matrix is built whose columns will be all spectra to be studied and rows will be formed by Raman shifts of the spectra. Due that all spectra are made up of 2330 Raman shifts, number of rows in this matrix is 2330. The columns depend on number of spectra in the study. Once data matrix is available, distance matrix must be determined. The elements of this matrix are all distances (calculated using the Euclidean metric) between the spectra. This distance matrix is used in the SPC algorithm.

Once clusters for the bank of cancer Raman spectra have been determined using SPC method, the most natural clusters are determined using the sequential procedure of Ott *et al* imposing a threshold value for *s*_*T*_, *s*_Θ_ = 0.5.

The calculation of the distance matrix, as well as the execution of the SPC algorithm and sequential procedure will take place on the Mat Lab platform.

## Results

### Breast cancer, leukemia and cervical cancer Raman spectra

**Fig 3:**
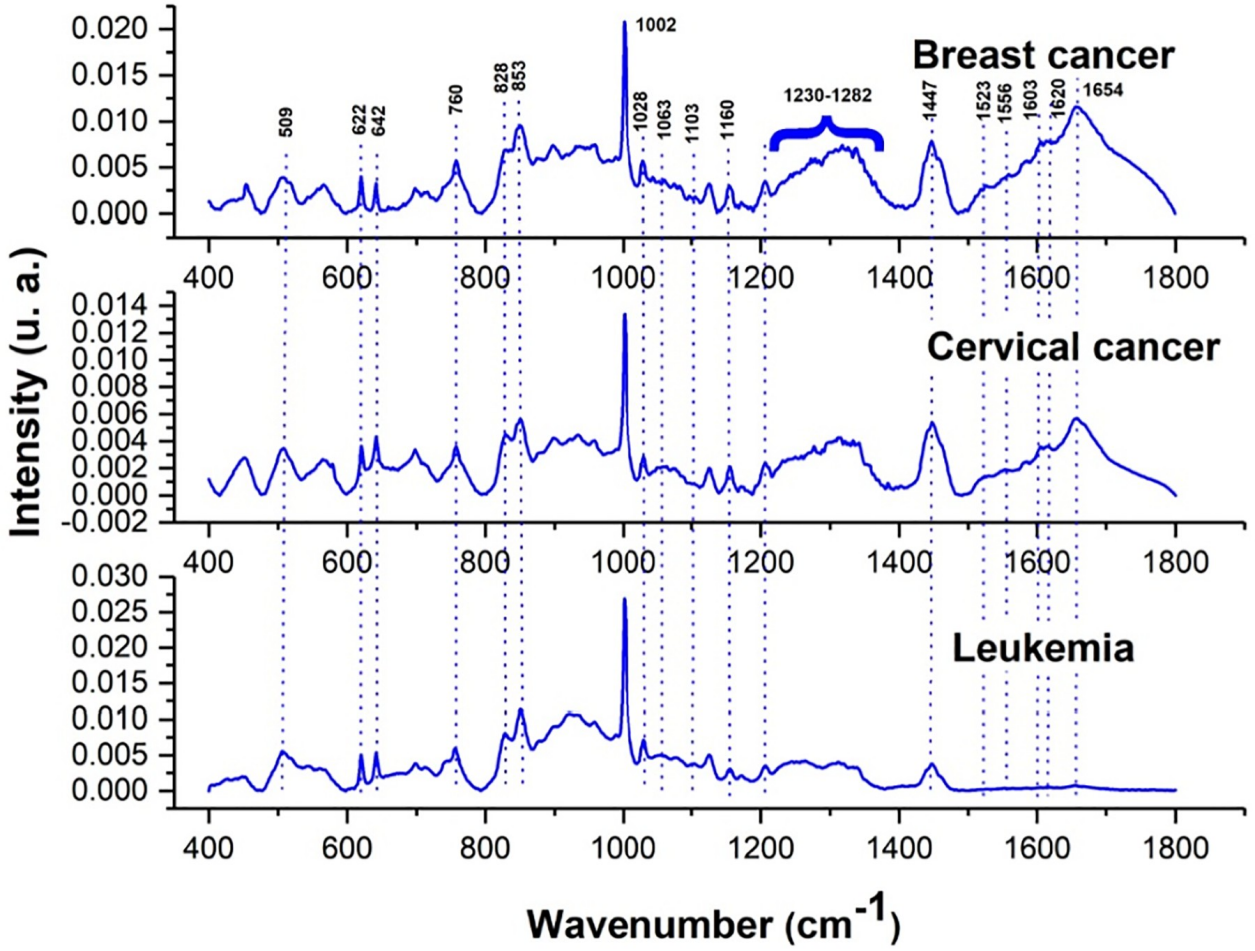
Average spectra of breast cancer, leukemia and cervical cancer samples.

Raw spectra were processed by carrying baseline correction using 5^*th*^ order polynomial fit for removing background fluorescence. A 2^*nd*^ order polynomial regression based on the Savitzky-Golay algorithm was used to perform smoothing removing shot noise from cosmic rays without affecting the signal shape. Finally spectra were normalized to the highest peak (phenylalanine band at 1002 *cm*^−1^). Once spectra was processed, SPC method was implemented. Fig 3 shows mean processed Raman spectra from breast cancer, leukemia and cervical cancer samples.

According to the literature [27], in these spectra can observe characteristic molecular vibration bands of this kind of samples localized at 622, 1002, 1028, 1063 and 1103 *cm*^−1^ corresponding to phenylalanine; 642, 828, 853 and 1603 *cm*^−1^ assigned to tyrosine; 1230-1282 *cm*^−1^ associated to amide III; 1447 *cm*^−1^ attributed to phospholipid; 1160 and 1523 *cm*^−1^ corresponding to *β* carotene; 509, 760, 1208, 1556 and 1620 *cm*^−1^ assigned to tryptophan; 714 *cm*^−1^ assigned to polysaccharides; 938 *cm*^−1^ attributed to skeletal str *α* and 1654 *cm*^−1^ for amide I.

However, some of molecules listed above were absent in some of the three spectra shown in Fig 3. Thus, for example, it is observed that peak corresponding to amide I protein was present in breast and cervical cancer spectra but completely absent in leukemia spectrum. This same fact occurs with the Raman bands that were close to it (1620, 1603, 1556 and 1523 *cm*^−1^). The peak 1447 *cm*^−1^ attributed to phospholipids also seems to vanish in leukemia spectrum. In contrast, the two shoulders assigned to amide III were present in leukemia, however, left shoulder seems to disappear in breast and cervical cancer spectra as well as the region around 938 *cm*^−1^ peak assigned to skeletal str *α*. On the other hand, band 714 *cm*^−1^ for polysaccharides seems to disappear only in cervical cancer spectrum. Finally, bulged region, 1040 − 1100 *cm*^−1^, attributed to phenylalanine, phospholipid (O-P-O and C-C) and *ν*s (C-C) phenylalanine in leukemia and cervical cancer spectra, seems to flatten in breast cancer spectrum.

### Discrimination between breast cancer, leukemia and cervical cancer samples using SPC model

In study, 160 breast cancer spectra, 150 cervical cancer spectra and 42 leukemia spectra were obtained. All spectra were measured in range 400 − 1800 *cm*^−1^ and each spectrum is composed of 2330 bands with their respective intensities.

In order to compare Raman spectra from breast cancer, leukemia and cervical cancer samples, spectra were processed as described in the previous section and constructed the data matrix, 2330 × 352, with first 160 columns corresponding to breast cancer spectra, next 150 columns to cervical cancer and the last 42 columns corresponding to leukemia spectra (see Table 1), and thus the distance matrix, 352 × 352.

**Fig 4:**
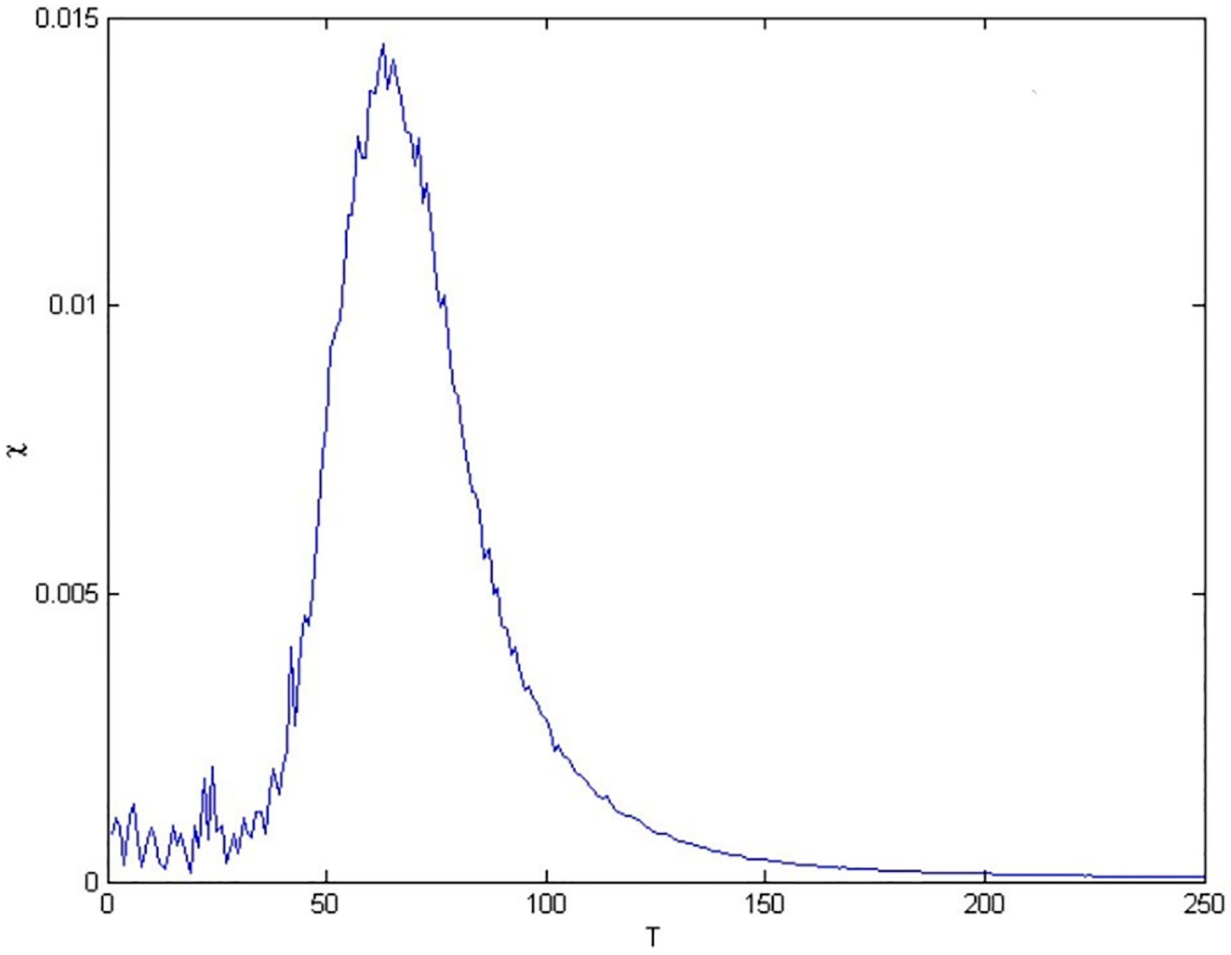
The magnetic susceptibility corresponding to breast cancer, leukemia and cervical cancer spectra, *χ*, as a function of temperature.

The optimal temperatures were collected for each network by determining the superparamagnetic phases where the first granulations of the networks took place. In order to locate this temperature, the susceptibility of the system, *χ*, is measured. In Fig 4 (where the scale denote the number of temperature steps of 0.001), at low temperatures can observe that all the points of the data remain within main cluster, as expected. When heated and the ferromagnetic-to-superparamagnetic phase transition temperature, indicated by the peak at *T* ≈ 0.062 in the susceptibility, is reached, main large cluster conformed by all Raman spectra from breast cancer, leukemia and cervical cancer begins to split and thus the hierarchical structure of data is uncovered, finding the physical clusters present.

**Fig 5:**
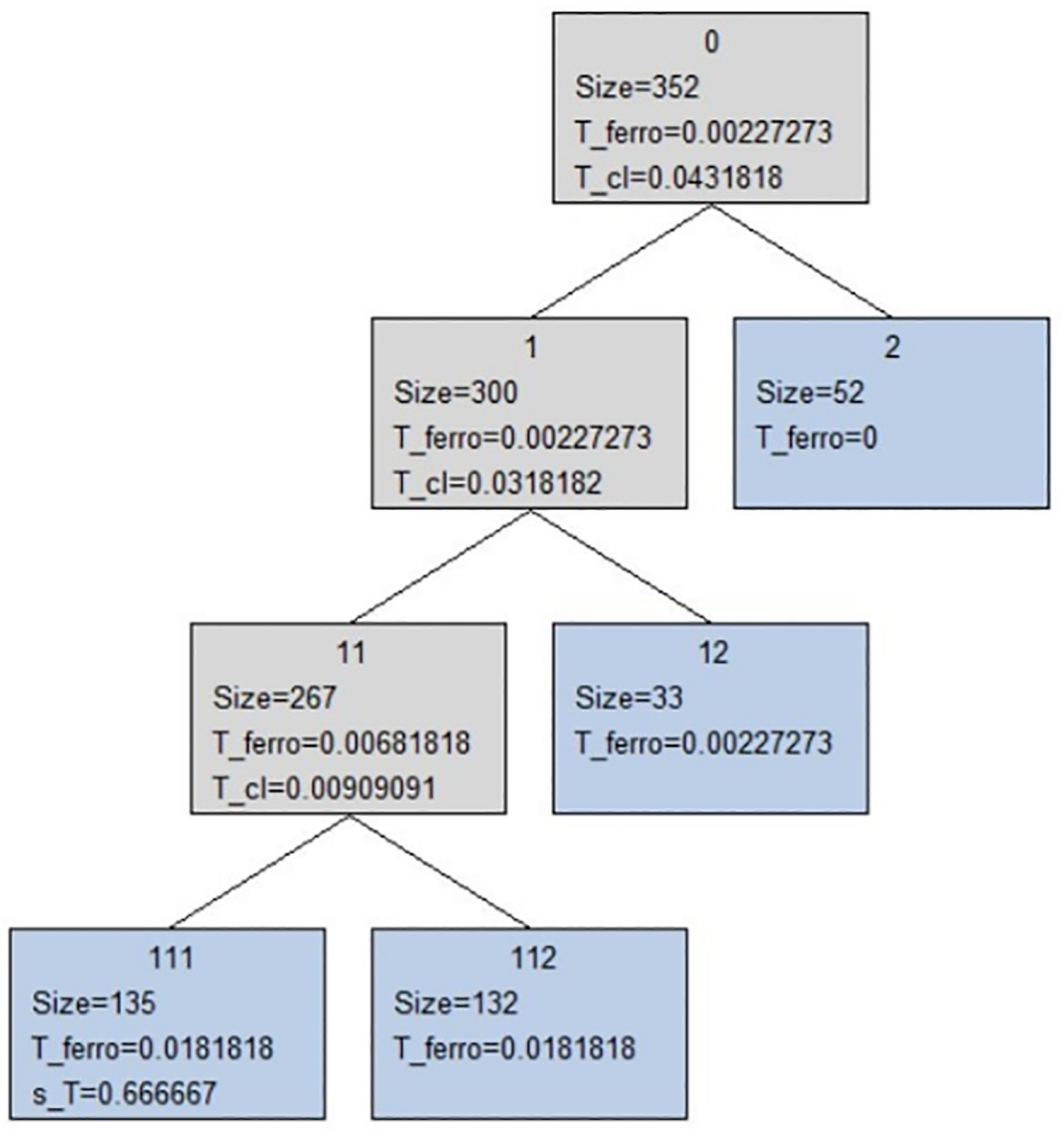
The tree diagram of breast cancer, leukemia and cervical cancer Raman spectra. The tree diagram provides the natural clusters (blue boxes) obtained in superparamagnetic phase.

In order to determine all natural clusters in which main cluster will be splitted, Stoop *et al* method [20] is applied to SPC model, resulting tree structure shown in Fig 5. In tree diagram, extracted natural clusters (blue boxes) form right branches and residual sets form left branches. In each box, size of the set, length of the ferromagnetic phase, *T*_*ferro*_, length of the *T-range* of the most stable cluster, *T*_*cl*_ (if any) and (optionally) paramagnetic transition temperature, *T*_*max*_, are reported. The stability values, *s*_*T*_, also are given for extracted natural clusters.

In Fig 5 and Table 2, observed that main cluster begins to split into cluster 1 with 300 elements and cluster 2 of size 52. They essentially remain stable in their composition until superparamagnetic-to-paramagnetic transition temperature is reached, expressed in a sudden decrease of *χ*, where the cluster 1 melt into the clusters, 1 1 (with size 267) and 1 2 (with size 33). Cluster 1 2 remains without substructure (natural cluster) while cluster 1 1 continues to split into natural clusters 1 1 1 and 1 1 2. Like cluster 1 2, cluster 2 remained unstructured so it is also a natural cluster.

**Table 2:** Clusters of breast cancer, leukemia and cervical cancer Raman spectra and their members. The spectra of the breast cancer, leukemia and cervical cancer clusters are indicated in magenta, green and yellow respectively.

Cluster	Size	*T*_*ferro*_	*T*_*cl*_	*s*_*T*_	Members
0	352	0.00227273	0.0431818		
1	300	0.00227273	0.0318182		
1 1	267	0.00681818	0.00909091		
1 1 1	135	0.0181818		0.666667	14, 16, 26, 34, 41, 42, 44, 45, 46, 54, 55, 57, 63, 81,
					161, 162, 163, 164, 165, 166, 167, 168, 169, 170, 171, 172, 173, 175, 176, 177, 178, 179, 180,
					181, 182, 183, 184, 185, 187, 188, 189, 190, 191, 192, 193, 194, 195, 196, 198, 202, 205, 206,
					207, 208, 209, 210, 211, 212, 213, 214, 215, 216, 217, 218, 222, 235, 236, 237, 238, 239, 240,
					241, 242, 243, 244, 245, 246, 247, 248, 250, 251, 252, 253, 254, 255, 256, 257, 258, 259, 260,
					261, 262, 263, 265, 266, 267, 269, 271, 272, 273, 274, 275, 276, 277, 278, 279, 280, 281, 282,
					283, 285, 286, 287, 289, 290, 291, 292, 293, 294, 295, 296, 297, 298, 299, 300, 301, 302, 303,
					304, 305, 306, 307, 309, 310,
					326
1 1 2	132	0.0181818			4, 5, 6, 7, 12, 13, 15, 17, 19, 22, 32, 33, 35, 37, 40, 43, 47, 49, 50, 51, 52, 53, 56, 58, 59, 60,
					62, 64, 68, 71, 72, 73, 74, 75, 76, 77, 78, 79, 80, 82, 83, 84, 85, 86, 87, 88, 89, 90, 94, 98, 101,
					102, 103, 104, 105, 106, 107, 108, 109, 110, 111, 132, 134, 135, 136, 137, 140, 141, 142, 144
					151, 152, 153, 154, 155, 156,
					174, 186, 197, 199, 200, 201, 203, 204, 249, 264, 268, 270, 284, 288, 308,
					311, 312, 313, 314, 315, 316, 317, 318, 319, 320, 321, 322, 323, 324, 325, 327, 328, 329, 330,
					331, 332, 333, 334, 335, 336, 337, 338, 339, 340, 341, 342, 343, 344, 345, 346, 347, 348, 349,
					350, 351, 352
1 2	33	0.00227273			10, 11, 18, 29, 30, 31, 38, 39, 67, 69, 95, 112, 113, 114, 115, 116, 117, 118, 119, 120, 121, 122,
					123, 131, 133, 139, 145, 147, 148, 149, 150, 158, 160
2	52	0			1, 2, 3, 8, 9, 20, 21, 23, 24, 25, 27, 28, 36, 48, 61, 65, 66, 70, 91, 92, 93, 96, 97, 99, 100, 124,
					125, 126, 127, 128, 129, 130, 138, 143, 146, 157, 159,
					219, 220, 221, 223, 224, 225, 226, 227, 228, 229, 230, 231, 232, 233, 234

Whether structure and spectra contained in main cluster are analyzed (see Tables 1 and 2), it is conclude that cluster 2 contains only spectra of breast cancer, so this cluster could significantly correspond breast cancer (indicated in magenta). Nevertheless, observing structure of cluster 1, it shows correct structure of Raman spectra from breast cancer, leukemia and cervical cancer patients, since it has a substructure of two clusters, 1 1 and 1 2, of which, second is a natural cluster whose elements (33) corresponds to the samples from breast cancer patients. The another cluster 1 1 shows a substructure of a natural cluster, 1 1 1, where a large number of spectra (120 of 135) correspond to samples from cervical cancer patients(indicated in green), and cluster 1 1 2 contains all spectra from the leukemia patients (indicated in yellow, except the 326 spectrum). Thus, clearly cluster 1 corresponds to leukemia and cervical cancer patients and cluster 2 corresponds to breast cancer patients; that is, as leukemia and cervical cancer clusters are inside same cluster 1 1 then it could be interpreted from biochemical point of view that leukemia seems to have a closer relationship with cervical cancer than with breast cancer.

On the other hand, substructures, clusters 2 and 1 2, could imply the existence of two types of breast cancer spectra each one corresponding to a stage of breast cancer, however, this cannot be confirmed by lack of clinical information concerning breast cancer stage of each patient, only known that patients were in advanced stages of breast cancer (II and III stages). In the same way, observing spectra contained in the clusters 1 1 1, 1 1 2 and 2, it could indicate the different stages of cervical cancer, however, the clinical information about cervical cancer stages is also unknown. In addition, within cluster 1, a whole cluster (cluster 1 2) containing only breast cancer spectra is observed (see Tables 1 and 2), so that it could show the biochemical relationships between the cancers, which as already indicated, it was reported by Qiao *et al* [7], Valentini *et al* [11] and Kan *et al* [10], who used techniques and methodologies different from the used in this study.

This same analysis was carried out by analyzing by pairs, that is, leukemia with breast cancer, leukemia with cervical cancer and breast and cervical cancer were studied separately. The results (not presented in this paper) show tree structures, with their respective spectra, in full agreement with the result presented in this work.

In SPC method, *χ* reflects reordering of clusters during the iteration process of the Markov chain, and a high degree of reordering yields a high *χ* indicating breakages of larger pieces into smaller ones occurring during a phase transition. In our case, SPC detected four natural clusters in data bank of Raman spectra labeled in tree diagram as 2, 1 2, 1 1 1 and 1 1 2 and whose members are showed in Table 2, where each member indicates the number of column in the data matrix, ie, spectrum number of one serum sample from one given patient (see Table 1).

Thus, SPC seems to provide the right classification of spectra, which allowed discriminating easily between types of cancer, so as information about biochemical type relationships between them.

### Comparison of the SPC and PCA results

In this section, standard method for analyzing Raman spectra will be applied to discriminate between the types of cancers and it will show that these results are in accordance with results obtained using the novel SPC method.

The PCA method is a method which is used to describe a data set in term of a new set of variables known as principal components (*PC*), allowing reflecting the greatest differences between the elements of the original data set. In PC space, large data clusters are observed, where greater similarities exist between elements of each cluster and non similarities between elements of different clusters. In general, distances between the clusters are increased applying linear discriminate analysis (LDA) method, allowing defined better the clusters found using PCA and thus know with greater probability elements of each group.

**Fig 6:**
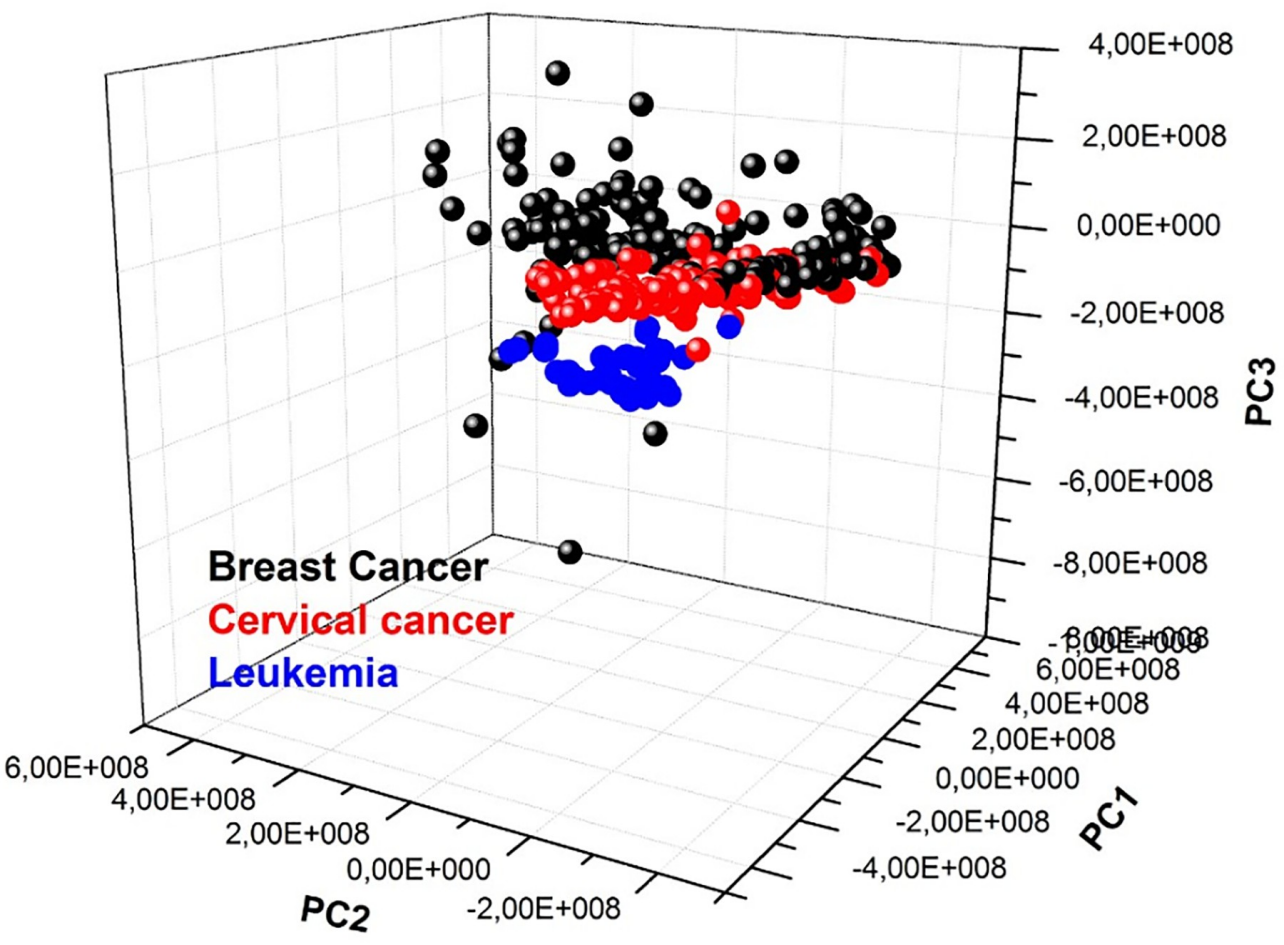
Scatter plot of breast cancer, leukemia and cervical cancer samples.

Fig 6 shows the PCA of all spectra of breast cancer, leukemia, cervical cancer. It can be observed that there are greater similarities between breast and cervical cancer than between these cancers and leukemia (as previously mentioned), however, an aspect that had not been appreciated was the fact that cervical cancer shows greater similarities with leukemia than with breast cancer. This fact is in complete agreement with SPC result since it reported that clusters associated to the leukemia and cervical cancer patients were inside same cluster 1 1. It is also observed that the three cancers share spectra, which could mean that the three cancers maintain a biochemical relationship with each other as was reported by Qiao *et al* [7], Valentini *et al* [11] and Kan *et al* [10].

Similarly, this analysis was carried out by analyzing separately, leukemia with breast cancer, leukemia with cervical cancer and breast and cervical cancer. The results (not presented in this paper) were in agreement with the result analyzing the three cancers as it is shown in Fig 6.

**Fig 7:**
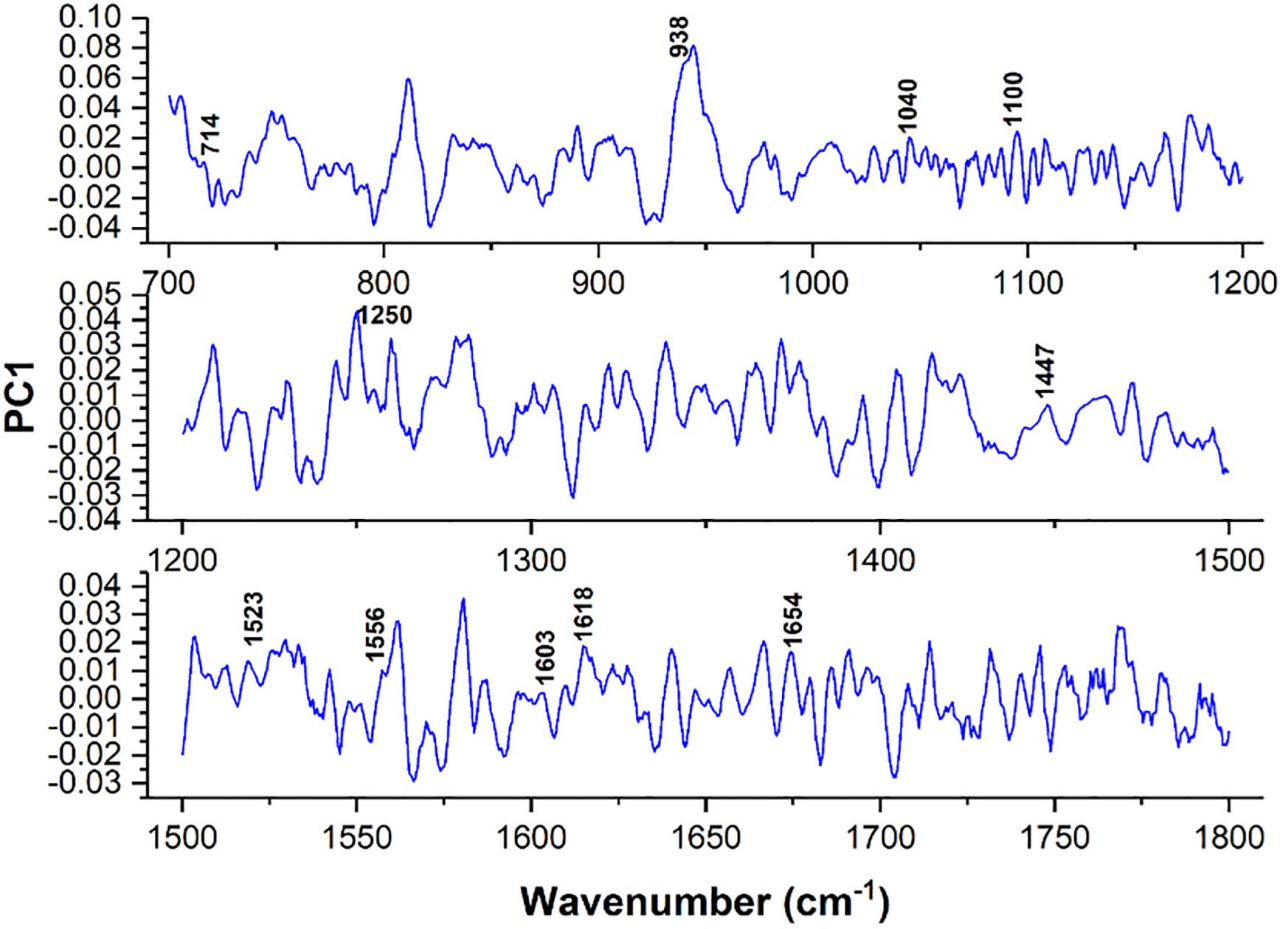
Plots of the first principal component as a function of the wavenumber.

In PCA, in order to bring out more clearly differences in spectral profiles, differences between the spectra could be computed by plotting the firsts principal components as a function of wavenumber. According to main characteristics of PCA, major differences between clusters are represented by positions (or wavenumber) of peaks with higher intensity. In this work, positions of the highest peaks of the *PC*1 principal component (see Fig 7) represent the natural biochemical differences among cancers (mainly peak 1250 *cm*^−1^ and region 1500-1800 *cm*^−1^), which are in complete agreement with spectral differences observed in Fig 3 and discussed at the beginning of this section.

Thus, clearly there is a excellent agreement between PCA and SPC results obtained by applying both methods to Raman spectra of the blood serum samples from breast cancer, leukemia and cervical cancer patients.

PCA algorithms were also implemented in Mat Lab commercial software.

## Conclusions

In this work, discrimination between breast cancer, leukemia, cervical cancer based on blood serum samples Raman spectroscopy and SPC method was studied. According to mean Raman spectra of breast cancer, leukemia and cervical cancer samples, peak associated to amide I (1654 *cm*^−1^) and one of two shoulders assigned to amide III (1230-1282 *cm*^−1^) allowed discriminating breast and cervical cancer from leukemia; the band 714 *cm*^−1^ (polysaccharides), the cervical cancer from leukemia and breast cancer and bulged region, 1040 − 1100 *cm*^−1^ (phenylalanine, phospholipid), breast cancer from the leukemia and cervical cancer. The tree structure obtained using SPC method also allowed concluding that although these degenerative diseases seem to be very different from each other, there are some aspects of biochemical type that allowed establishing relationships between them. This tree structure showed that clusters corresponding to leucemia and cervical cancer spectra belonged to a larger cluster, which together with another one corresponding to breast cancer, make up the largest cluster formed by all the spectra, ie, leukemia maintains greater similarities with cervical cancer but without the loss of relationships between the three cancers. Motivated by researches of genetic and biochemical type about cancer, reporting relationships between certain forms of leukemia, breast, lung, colon and cervical cancer, in this work were studied similarities between cancers using alternative techniques to those already reported, hoping that the best knowledge of these similarities based on the same molecular origin could facilitate the comparison of therapeutic data bank suggesting that certain treatments could be performed with same chemotherapy drugs. Finally, as a cross check, the standard method to classify Raman spectra of breast cancer, leukemia and cervical cancer, PCA, was used and results were in complete agreement with result obtained by applying SPC method. Thus, preliminary results demonstrated that Super Paramagnetic Clustering method allows properly classifying Raman spectra and discriminate between breast cancer, leukemia and cervical cancer patients based on chemical composition of blood serum samples indicated by the bands in a Raman spectrum.
